# The mechanism of flavonoids from *Cyclocarya paliurus* on inhibiting liver cancer based on *in vitro* experiments and network pharmacology

**DOI:** 10.3389/fphar.2023.1049953

**Published:** 2023-02-03

**Authors:** Jinggang Mo, Yingpeng Tong, Junxia Ma, Kunpeng Wang, Yifu Feng, Liezhi Wang, Hao Jiang, Chong Jin, Junmin Li

**Affiliations:** ^1^ Department of General Surgery, Taizhou Central Hospital (Taizhou University Hospital), Taizhou University, Taizhou, Zhejiang, China; ^2^ School of Advanced Study, Taizhou University, Taizhou, China; ^3^ Zhejiang Provincial Key Laboratory of Evolutionary Ecology and Conservation, Taizhou University, Taizhou, China

**Keywords:** *Cyclocarya paliurus*, flavonoids, active ingredients, *in vitro* experiments, network pharmacology, liver cancer, apoptosis

## Abstract

**Introduction:** *Cyclocarya paliurus* (Batal.) Iljinsk., a subtropical tree belonging to the family Juglandaceae, is rich in polysaccharides, flavonoids, and terpenoids. It has important pharmacological effects such as lowering blood lipids, blood sugar, and blood pressure. However, little has been discerned regarding anti tumor effects and their potential mechanisms.

**Method:** **In vitro** cell culture experiments were used to test the effect of *C. paliurus* total flavonoids (CTFs) extract on apoptosis mechanisms in HepG2 cells. Network pharmacology was applied to further explore the effects of CTFs on liver cancer as well as the mechanisms through which these effects might be achieved. Both 3 hydroxyflavone and luteolin were randomly selected to verify the effect on inducing apoptosis and inhibiting the proliferation of HepG2 cells.

**Results and Discussion:** Network pharmacological analysis was applied to these 62 compounds and their targets, and 13 flavonoids were further screened for their potential anti liver cancer activity. These 13 flavonoids included: tangeretin, baicalein, 7,3′-dihydroxyflavone, velutin, 3-hydroxyflavone, chrysin, kumatakenin, tricin, luteolin, chrysoeriol, apigenin, pinocembrin, and butin. Together, these flavonoids were predicted to interact with AKT1, MAPK3, PIK3CA, EGFR, MAP2K1, SRC, IGF1R, IKBKB, MET, and MAPK14. It was predicted that the inhibitory effect on hepatocellular carcinoma would be accomplished by regulation of core proteins relating to such KEGG pathways as cancer, PI3K-Akt, proteoglycans in cancer, microRNAs in cancer, and endocrine resistance via core target proteins. Both 3-hydroxyflavone and luteolin were demonstrated to induce apoptosis and inhibit the proliferation of HepG2 cells. Our study provides scientific evidence supporting the use of CTFs for the treatment of liver cancer.

## 1 Introduction

Liver cancer represents a major public health concern, with the fifth highest incidence rate and the second highest fatality rate globally across all cancer types (Sun et al., 2017; Zhou et al., 2018; Liu et al., 2020). Traditional Chinese medicine (TCM) has been used to treat disease in China for over two thousand years (Tang et al., 2008), and clinical studies have provided evidence that TCM can provide positive therapeutic effects in the prevention and treatment of liver cancer (Chen et al., 2016; Tang et al., 2020). For example, the extract of *Brucea javanica* seeds was shown to eliminate sphere formation in cancer stem cells and inhibit tumor-initiating behavior in hepatocellular carcinoma cells (Ren et al., 2011; Chen et al., 2016). Identifying effective components from TCM to prevent and treat liver cancer is becoming an urgent issue (Liu et al., 2020).

Network pharmacology, a new branch of pharmacology based on systems and network biology, focuses on revealing the complex network of relationships between drug, target, and disease, systematically observing the intervention and influence of drugs on the disease network and providing insight into the molecular mechanism of drug therapies (Zhao and Iyengar, 2012; Kibble et al., 2015). Recently, network pharmacology has generated a great deal of interest for its potential in elucidating the mechanistic details behind myriad TCM treatments for complex diseases such as cancer, cardiovascular disorders, and diabetes (Duan et al., 2019; Wufuer et al., 2022). A recent study by Sun W. et al. (2021) predicated potential signaling pathways and active ingredients of the TCM root tuber *Drynariae Rhizoma via* network pharmacology, and found that flavonoids in *Rhizoma Drynariae* improved the outcomes of large bone defects in mice by activating the mitogen-activated protein kinase (MAPK) signaling pathway. Another study by Liu et al. (2020) used network pharmacology to explore the anti-tumor effect of total flavonoids extracted from the TCM herbal formulation Pien-Tze-Huang, on liver cancer, concluding that the mechanism underlying their effect on liver cancer involved a synergy of effects from multiple components on multiple targets and multiple pathways.

Flavonoids, a group of metabolites characterized by a diphenylpropane structure (C6-C3-C6), are widely distributed in plants, including fruits and vegetables (López-Lázaro, 2009). Flavonoids extracted from plants used in TCM have been shown to exert obvious *in vitro* and *in vivo* anti-tumor activities in such contexts as acute myeloid leukemia (Hirano et al., 1994), lung cancer (Yang et al., 2000), melanoma (Caltagirone et al., 2000), and others. A few studies showed that flavonoid monomers and their derivate flavonoids also have significant inhibitory effects on liver cancers (Eaton et al., 1996; Chen et al., 2009; Quan et al., 2013; Kasala et al., 2015). For example, the derivates of chrysin extracted from *Passiflora caerulea* can inhibit the migration, invasion, proliferation, self-renewal, and stemness of liver cancer stem cells (Tang et al., 2020). Recently, several studies have even applied network pharmacology to predict the potential inhibitory effects of plant flavonoids on liver cancer (Liu et al., 2020; Sun W. et al., 2021).

*Cyclocarya paliurus* (Batal.) Iljinsk. is endemic to China. The leaves of *C. paliurus* have long been used in traditional Chinese medicine for their anti-diabetic, anti-bacterial, and anti-cancer properties (Xie et al., 2016; Wu et al., 2017). The polysaccharide from *C. paliurus* has been shown in modern studies to exert antiproliferative effects on various tumor cell lines (Luo et al., 2015; Shinbo et al., 2015; He et al., 2018). Flavonoids are the most common compounds in the leaves of *C. paliurus* (Liu Y. et al., 2016), with a recent study identifying a total of 188 different flavonoids in the leaves of *C. paliurus via* LC-MS-MS (Sheng et al., 2021). *C. paliurus* total flavonoids (CTFs) have been shown to significantly lower blood sugar, blood fat, and blood pressure, as well as to improve immunity (Liu et al., 2018; Sun C. et al., 2021). Moreover, CTFs can effectively reduce oxidative stress and protect mice from acute liver injury (Xie et al., 2018). However, little has been known about the pharmacological effect of CTFs on liver cancer. In this study, we tested the effect of CTFs on apoptosis mechanisms in HepG2 cells. We then applied network pharmacology to further explore the effects of CTFs on liver cancer as well as the mechanisms through which these effects might be achieved.

## 2 Materials and methods

### 2.1 Plant samples

Leaves of *C. paliurus* were collected from Zhuzhang Village, Longquan City, Lishui City, Zhejiang Province, China (E118°48′28″, N28°5′57″). A portion of leaves were frozen in liquid nitrogen immediately after harvest and transferred to a −80°C freezer until metabolomic analysis. The remaining leaves were dried at 70°C to a constant weight and used for the extraction of total flavonoids.

### 2.2 Preparation of CTFs and determination of the total flavonoids content

Dried leaves were ground and sieved through a 40-mesh sieve. Powders were ultrasonic soaked in 70% ethanol (1:5, w/v) for 30 min and filtered with filter paper. This process was repeated a total of three times, and supernatants were combined and concentrated in a vacuum rotary evaporator at 70°C. About 400 g extract was suspended in 1 L H_2_O and the solution was extracted with 250 mL ethyl acetate for six times. The ethyl acetate extracts were further subjected to polyamide macroporous adsorption resin and the CFTs were collected for downstream *in vitro* experiments. The total flavonoids content were determined by AlCl_3_ colorimetry according to the method described by Sheng et al. (2021). Rutin was used as a standard and the determinations were carried out for three replicates.

### 2.3 *In vitro* experiments of CTFs

#### 2.3.1 Cell culture

HepG2 cells were purchased from the National Collection of Authenticated Cell Cultures, Shanghai, China and STR DNA typing (Dirks and Drexler, 2011) was used to identify this cell line (results were provided in (results were provided in Supplementary Material S1). High-glucose DMEM medium supplemented with 10% fetal bovine serum (FBS) (Universal Biotech Co., Ltd., Shanghai, China) and 1% penicillin/streptomycin (Sangon Biotech Co., Ltd., Shanghai, China) was used to culture the cells in a humidified atmosphere of 95% air and 5% CO_2_ at 37°C in a cell culture incubator (Memmert GmbH + Co. KG, Schwabach, Germany). Media was replaced at 2-day intervals.

#### 2.3.2 CTFs inhibit the proliferation of HEPG2 cell

Cellular proliferation was assessed using the MTT Cell Proliferation and Cytotoxicity Assay Kit (Universal Biotech Co., Ltd., Shanghai, China). HepG2 cells were cultured in 96-well plates for 24 h and treated with 0 mg/L, 1.51 mg/L, 3.02 mg/L, 7.55 mg/L, 15.1 mg/L, 30.2 mg/L, 45.3 mg/L, and 90.6 mg/L of CTFs (resolved in DMSO) and incubated for another 48 h. All treatment concentrations, including the control 0 mg/L, contained equal amounts of DMSO. Media was then replaced with fresh media that had been mixed with 10% (V/V) MTT (5 mg/mL). Wells were incubated with formazan solvent for an hour at 37°C before absorbance was detected at OD570 and OD690 using a Multimode Microplate Reader (TECAN Spark, Männedorf, Switzerland). Relative Cell Activity and the Inhibition Rate of CTFs were calculated as follows. Relative Cell Activity = (OD570_s_–OD690_s_)/(OD570_0_–OD690_0_) × 100%, where OD570_s_ and OD690_s_ were absorbance of the tested sample at 570 nm and 690 nm, respectively, and OD570_0_ and OD690_0_ were absorbance of the negative control at 570 nm and 690 nm, respectively. Inhibition Rate = (1- Relative Cell Activity) × 100%.

#### 2.3.3 CTFs promote apoptosis in HEPG2 cells

Cellular apoptosis was assessed using the Apoptotic and Necrotic Detection Kit Triple Fluorescence Color (Sangon Biotech Co., Ltd., Shanghai, China). As for flow cytometric analysis (BD FACS Aria II, New Jersey, America), HepG2 cells were cultured in 12-well plates for 48 h and then incubated with 0 mg/L, 7.55 mg/L, 15.1 mg/L, and 30.2 mg/L of CTFs (resolved in DMSO) for 24 h. Next, cells were collected, centrifuged at 300 × g for 5 min, and washed twice with PBS at room temperature. Cells were resuspended with 400 μL 1 × assay buffer at the concentration of 1 × 10^6^ cells/mL, and then incubated with Apopxin Green (4 μL), 7-AAD (2 μL), and CytoCalcein Violet 450 (2 μL) at room temperature for 20 min in the dark, according to the manufacture’s instructions. Events in the upper left corner of the figures, marked Q1, represent dead cells and naked nuclei. Events in the upper right corner, marked Q2, represent late-stage apoptotic and necrotic cells. Events in the lower left corner, marked Q3, represent normal cells. Events in the lower right corner, marked Q4, represent early-stage apoptotic cells. Apoptotic rate was calculated by summing the percentages of Q2 + Q4. As for Confocal Laser Scanning Microscope (CLSM) analysis (IX83-FV3000, Olympus Corporation, Tokyo, Japan), HepG2 cells were cultured in 4-well glass bottom plates specially designed for CLSM applications (Xinyou Technology Co., Ltd., Hangzhou, China). Treatment with CTFs and cell staining with the Apoptotic and Necrotic Detection Kit, which was performed as described above.

### 2.4 Network pharmacology analysis of CTFs

#### 2.4.1 Compound database construction

The flavonoids used for network pharmacology analysis in this study were collected from our previous work (Sheng et al., 2021). Briefly, the fresh leaves of *C. paliurus* were freeze-dried and extracted for secondary metabolite analysis by HPLC/MS/MS and its accompanying self-built database by Metware Biotechnology Co., Ltd. (Wuhan, China). Among 188 flaovnoids in the leaves of *C. paliurus*, a total of 114 CTFs were verified to have clear chemical structures which were obtained from Pubchem databases (https://pubchem.ncbi.nlm.nih.gov/). If chemical structures could not be found there, original research articles were reviewed and the structures were drawn by ChemDraw 16.0 software (https://www.chemdraw.com.cn/xiazai html).

#### 2.4.2 Protein target database construction

CTF structures were saved in SMILES format and searched in SwissADME database (http://www.swissadme.ch/) to screen compounds used in the present work based on Lipinski’s Rule of Five. Targets of these filtered compounds were then predicted using SwissTargetPrediction (http://www.swisstargetprediction.ch/). Duplicates were deleted after combining the targets of each component, and potential targets were obtained through network topology analysis.

#### 2.4.3 Anti-liver cancer target database construction

Relevant targets for the inhibition of liver cancer proliferation were identified by searching the MalaCards database (https://www.malacards.org/) with the following keywords: “Hepatocellular Carcinoma.” The results were summarized and sorted according to the gene names. Common targets between the disease database and the compound database were indicated as potential targets of CTFs responsible for their therapeutic effects in the context of liver cancer.

#### 2.4.4 GO and KEGG pathway enrichment analysis

Potential targets were imported into the DAVID 6.8 database (https://david.ncifcrf.gov/) to perform GO and KEGG pathway enrichment analysis. “OFFICE_GENE_SYMBOL” and “*Homo sapiens*” were selected. The top 10 enriched GO entries and top 30 enriched KEGG pathways that met the criteria of *p* < 0.01 were selected and uploaded to the OmicShare (http://www.omicshare.com/tools/) cloud platform for data visualization, where the size of the bubble represents the number of genes in the pathway, and the color of the bubble represents the significance of enrichment.

#### 2.4.5 Construction of core component-target-pathway network

The integrated network of component-target-pathway was constructed using Cytoscape 3.9.0 to show the relationships among the active ingredient compounds, the target proteins, and the pathways. The topology characteristics of the network were evaluated.

### 2.5 Molecular docking of active ingredient-key target

To further verify the reliability of the target prediction results, the top 10 scoring targets in the “component-target-pathway” network were selected for molecular docking analyses using Auto dock vina to confirm their expected binding capabilities with their cognate CTF ligands (Trott and Olson, 2010).

### 2.6 *In vitro* verification of predicted compounds

3-hydroxyflavone and luteolin were purchased from DESITE Biotechnology Co., Ltd., Chengdu City, Sichuan Province, China. 3-hydroxyflavone and luteolin were randomly selected and *in vitro* experiments to explore the specific effects were performed according to the methods described above for whole CTFs.

### 2.7 Statistical analysis

Statistical analysis was carried out using SPSS 19.0. One-way analysis of variance (ANOVA) was applied to measure the significance of comparisons between groups after homogeneity of variance was confirmed. Tukey’s *post hoc* HSD test was used in case of significance of the ANOVA test. Differences were considered statistically significant when the *p*-value was less than 0.05. All data are shown as mean ± standard deviation.

## 3 Results

### 3.1 Effects of CTFs on HepG2 cells

**FIGURE 1:**
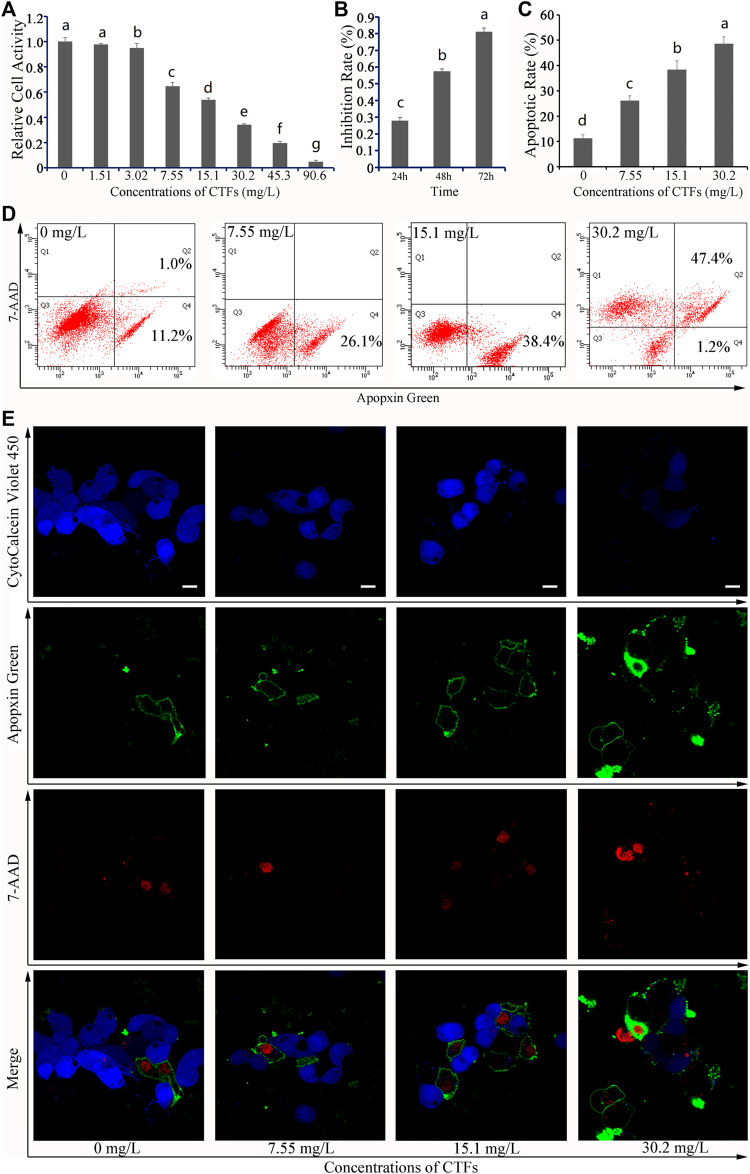
Effects of *Cyclocarya paliurus* total flavonoids (CTFs) on HepG2 cells. **(A)** Relative cell activity, **(B)** Inhibition rate, and **(C)** Apoptotic rate. Data are expressed as the mean ± SEM (*n* = 3). Different small letters indicate extremely significant differences between two treatments (*p* < 0.01). **(D)** Representative FACS plots of cells treated with different concentrations of CTFs and stained with Apopxin Green and 7-AAD to identify apoptotic cells in the early and late stages, respectively. From left to right, the concentration of CTFs was 0 μg/mL, 7.55 μg/mL, 15.1 μg/mL, and 30.2 μg/mL. Data are representative of four independent experiments. **(E)** Representative CLSM images of HepG2 cells treated with different concentrations of CTFs and stained with CytoCalcein Violet 450 (in blue color, representing live cells), Apopxin Green (in green color), 7-AAD (in red color). From left to right, the concentration of CTFs was 0 μg/mL, 7.55 μg/mL, 15.1 μg/mL, and 30.2 μg/mL. Data are representative of four independent experiments.

The total flavonoids in CTFs was 81.82% ± 0.36%. The results of the MTT experiment showed that the cell activity of HepG2 cells decreased as CTFs concentration increased. When the concentration of CTFs reached 90.6 mg/L, the cell activity of HepG2 cells was only 4.94% relative to the DMSO-only control (Figure 1A). The IC50 value of the inhibitory effect of CTFs on HepG2 cells was 10.79 mg/L, indicating an effective treatment concentration ranged from 0 to 30.2 mg/L in this context.

After 24, 48, and 72 h of treatment with CTFs, the inhibition rate of 15.1 mg/L CTFs on HepG2 cells was 28.03%, 57.46%, and 93.46%, respectively (Figure 1B), indicating that treatment with 48 h would give a moderate effect. Therefore 48 h was selected for further study.

After 48 h of treatment with 0 mg/L, 7.55 mg/Ll, 15.1 mg/L, and 30.2 mg/L of CTFs, the apoptosis rate of HepG2 cells was 11.2% ± 1.4%, 26.1% ± 1.9%, 38.4% ± 3.5%, and 76.6% ± 2.8%, respectively, indicating that CTFs could promote the apoptosis of HepG2 cells in a dose-dependent manner (Figures 1C, D). Based on the CLSM analysis, most of the cells in the 0 μM group were live cells stained with blue, while only two or three cells were stained with green and/or red, indicating that the apoptotic cells in either the early or the late stage were very few (Figure 1E). In contrast, cells treated with 7.55 mg/L, 15.1 mg/L, and 30.2 mg/L CTFs were stained more and more weakly with blue and instead showed darker green and/or red, indicating that increased dosage of CTFs led to a corresponding increase of cells in the early and late stages of apoptosis, respectively (Figure 1E). Together, these results indicated that the inhibitory effect of CTFs on the growth of HepG2 cells was likely accomplished *via* induction of cellular apoptosis.

### 3.2 Screening of potential targets of *C. paliurus* flavonoids

After screening, a total of 62 compounds met the Lipinski rules (Supplementary Table S1), and a total of 354 target proteins were predicted through the SwissTargetPrediction Database. Interaction analyses of these two datasets yielded 64 common targets considered as potential anti-liver cancer targets of CTFs (Supplementary Table S2; Supplementary Figure S1).

### 3.3 GO and KEGG pathway enrichment of target proteins

**FIGURE 2:**
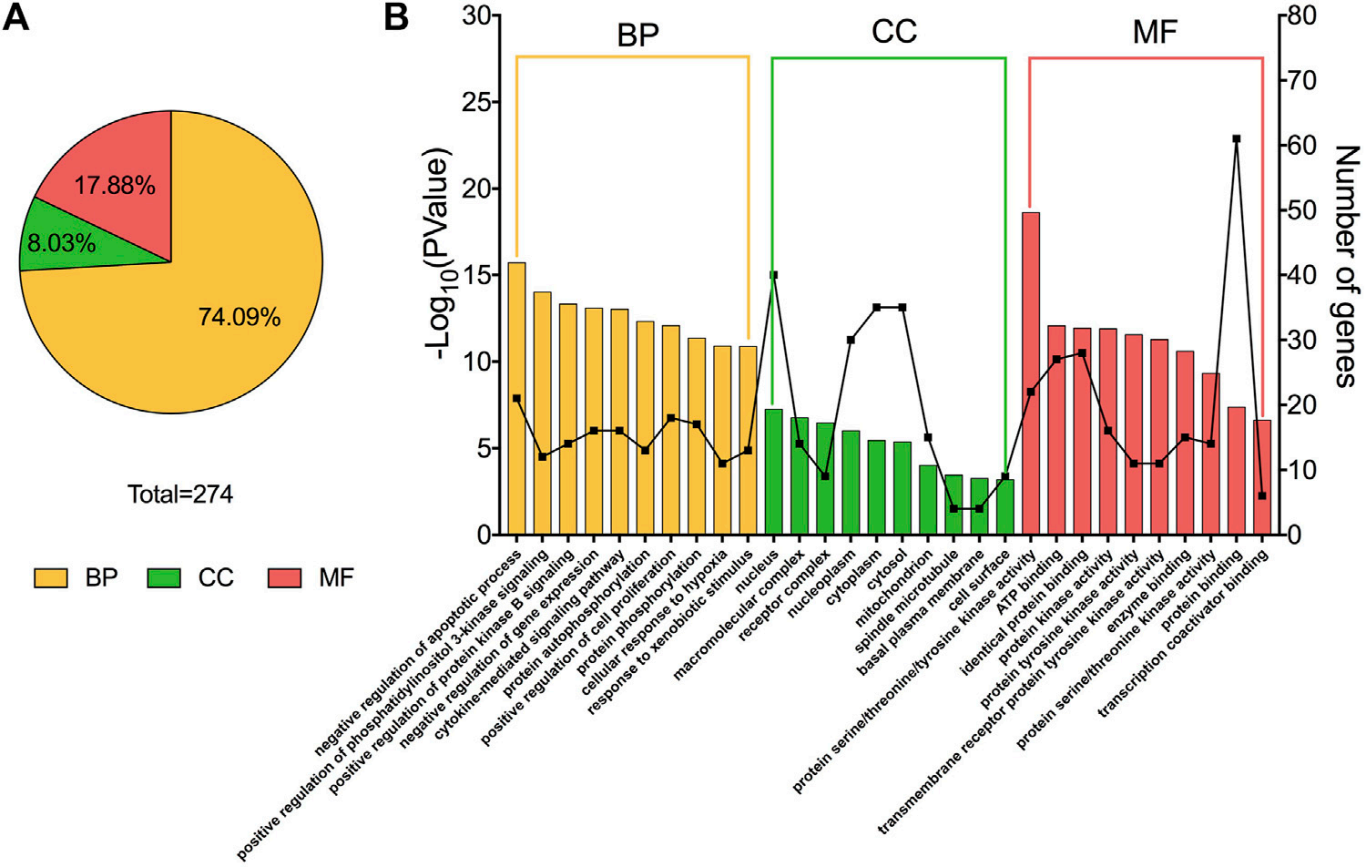
GO enrichment analysis results. **(A)** The percentage of biological process (BP), cellular component (CC), and molecular function (MF) entries in total GO entries (*p* < 0.05). **(B)** The top 10 entries of BP, CC and MF, included their gene number, ranked from left to right by −log10 (*p*-value).

GO enrichment analysis of target proteins indicate that the numbers of target proteins involved in the biological processes (BP), molecular functions (MF) and cellular components (CC) categories are 203 (74.09%), 49 (17.88%), and 22 (8.03%) (Figure 2A). The top 10 subcategories in BP, MF, and CC are shown in Figure 2B. In the BP category, target proteins were mainly involved in negative regulation of apoptotic processes. The CC category revealed that these proteins were most associated with the nucleus, and the MF category highlighted protein serine/threonine/tyrosine kinase activity as the most affected molecular function by CTFs. Together, these results provide a possible window into the pathways and processes through which CTFs are likely to exert their inhibitory effects on hepatocellular carcinomas.

**FIGURE 3:**
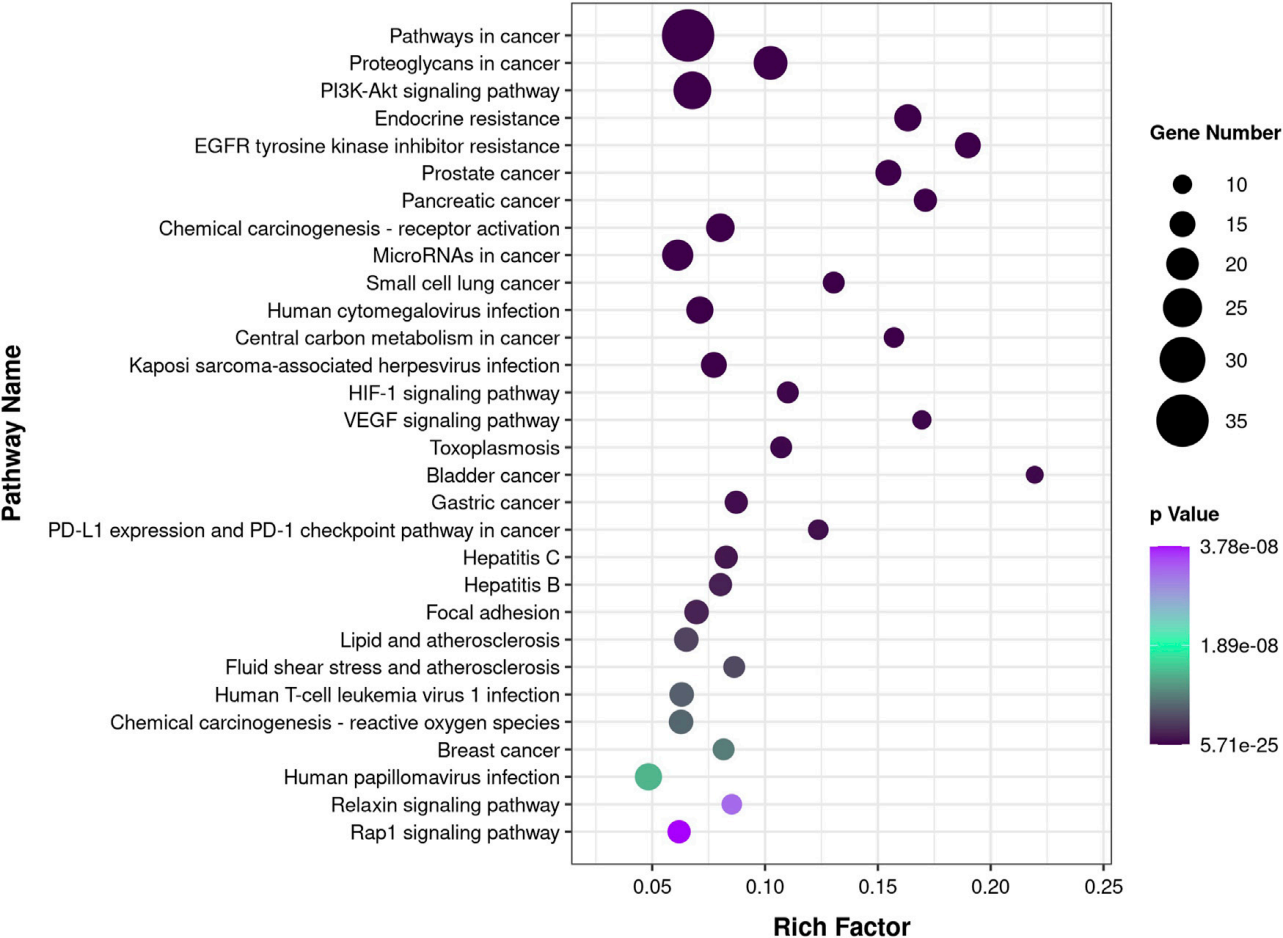
KEGG pathway enrichment analysis. The color of the circles is displayed in a gradient from purple to black according to the adjusted *p*-value for each KEGG pathway, with black indicating a more significant *p*-value. The size of the circles corresponds to the number of genes affected in KEGG each pathway.

According to the results of enrichment analysis based on KEGG pathway analysis (Supplementary Table S3), the top 30 pathways related to those core targets are shown in Figure 3. The top five pathways were identified as cancer (hsa05200, 54.69%), PI3K-Akt signaling pathway (hsa04151, 37.5%), proteoglycans in cancer (hsa05205, 32.81%), microRNAs in cancer (hsa05206, 29.69%), and endocrine resistance (hsa01522, 25%). These results suggest that CTFs may exert their inhibitory effects on hepatocellular carcinoma by regulating the above mentioned pathways *via* core target proteins.

### 3.4 The component-target-pathway network

The component-target-pathway network was constructed to visualize all interactions between target proteins and anti-liver cancer-related pathways. Based on the previous KEGG enrichment analysis, a component-target-pathway network was generated by connecting compounds, targets and pathways (Supplementary Table S3; Supplementary Figure S2). This network included 229 nodes (54 active compound nodes, 59 composite target protein nodes, and 116 pathway nodes) as well as 7,258 edges.

**FIGURE 4:**
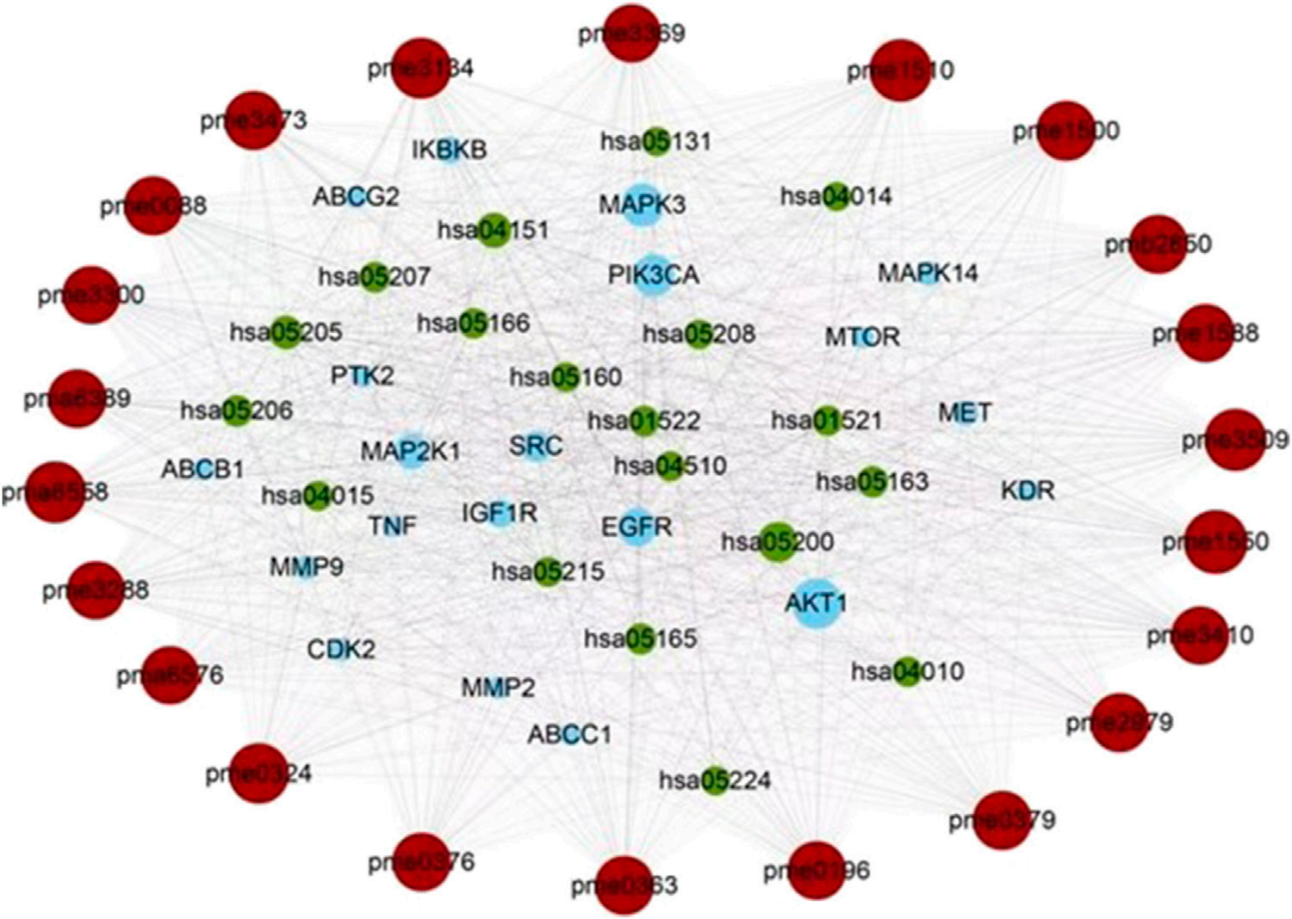
The core of the component-target-pathway network. Nodes filled with brown, blue, and green represent the active ingredients, targets, and pathways, respectively. The size of the node represents the degree value. Lines represent the relationships between the compounds, targets, and pathways.

**FIGURE 5:**
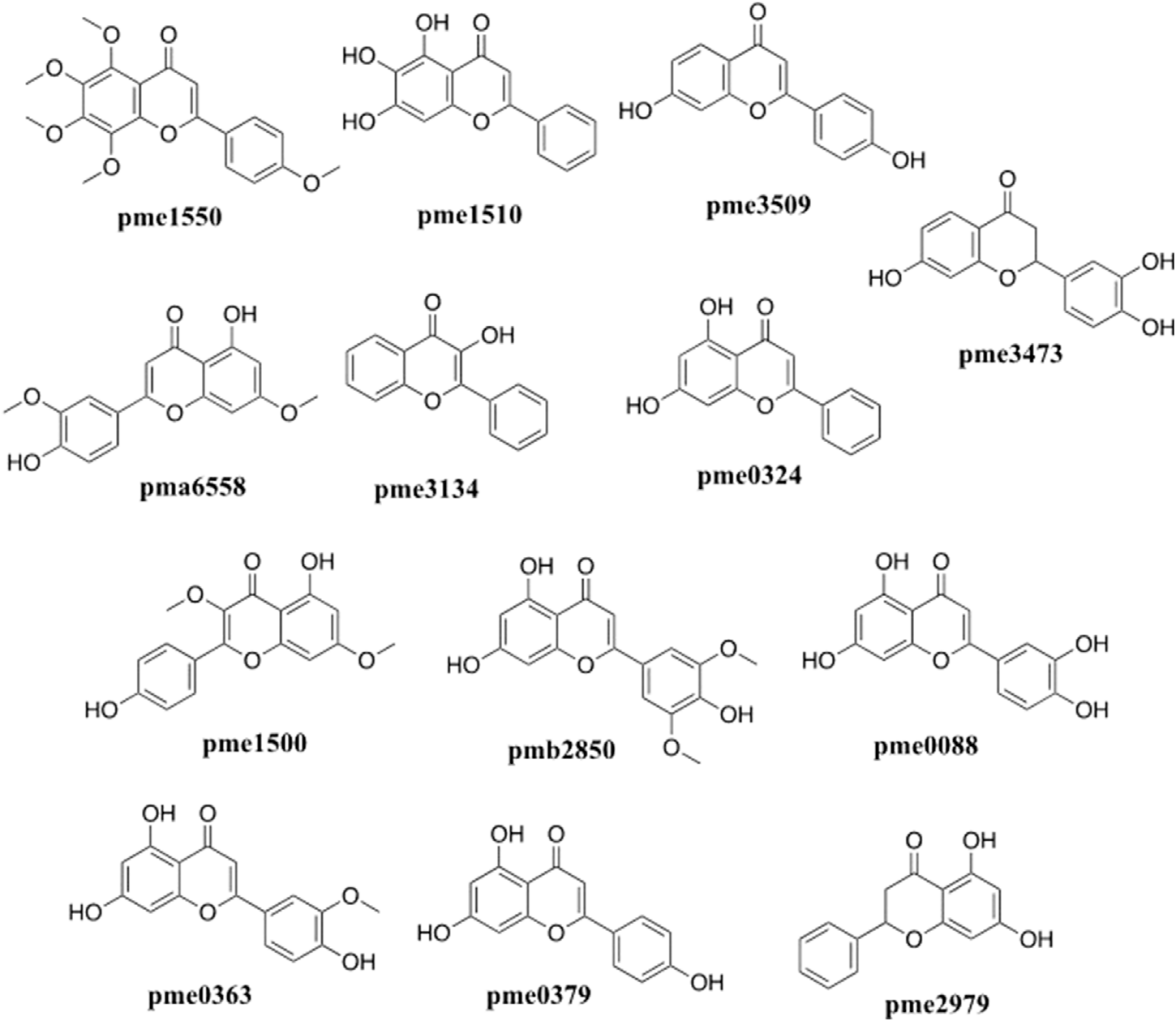
Chemical structures of the potential flavonoids compounds of *C. paliurus* with anti-liver cancer effects screened by network pharmacology analysis.

The network analysis showed that the median value of betweenness centrality and closeness centrality was greater than the median with high degree for a total of 10 targets, including AKT1, MAPK3, PIK3CA, EGFR, MAP2K1, SRC, IGF1R, IKBKB, MET, and MAPK14 (Table 1). These target proteins can be considered as core targets for the treatment of CTFs on liver cancer (Figure 4). Among these KEGG pathways, those related to cancer (hsa05200), PI3K-Akt signaling pathway (hsa04151), proteoglycans in cancer (hsa05205), microRNAs in cancer (hsa05206), human cytomegalovirus infection (hsa05163), human papillomavirus infection (hsa05165), endocrine resistance (hsa01522), chemical carcinogenesis—receptor activation (hsa05207), EGFR tyrosine kinase inhibitor resistance (hsa01521), MAPK signaling pathway (hsa04010), and human T-cell leukemia virus 1 infection (hsa05166) have been shown to have clear links with liver cancer occurrence. The structures of 13 potential active compounds were shown in Figure 5. The degree values of these compounds were very close and ranged from 133 to 144 due to the similar chemical structures.

**TABLE 1:** The top ten active ingredients, targets, and pathways that affect the entire network with high median values of betweenness centrality and closeness centrality.

No.	Compound	Degree	Target	Degree	Pathway	Degree
1	Tangeretin (pme1550)	144	AKT1	113	hsa05200	89
2	Baicalein (pme1510)	141	MAPK3	96	hsa04151	78
3	7,4′-Dihydroxyflavone (pme3509)	140	PIK3CA	93	hsa05205	74
4	Velutin (pma6558)	139	EGFR	85	hsa05206	70
5	3-Hydroxyflavone (pme3134)	137	MAP2K1	77	hsa05163	69
6	Chrysin (pme0324)	136	SRC	68	hsa05165	69
7	Kumatakenin (pme1500)	135	IGF1R	62	hsa01522	68
8	Tricin (pmb2850), Luteolin (pme0088), Chrysoeriol (pme0363), Apigenin (pme0379), Pinocembrin (pme2979), Butin (pme3473)	133	IKBKB	60	hsa05207	68
9			MET	56	hsa01521, hsa04010, hsa05166	67
10			MAPK14	53		

### 3.5 Molecular docking of active ingredient-key target

Except for MAPK3, for which the corresponding protein structure cannot be found, the remaining top 10 targets were analyzed by molecular docking to assess their binding capabilities. The docking results showed that all binding energies were lower than −5 kcal/mol, indicating the top 10 active compounds have reasonably good binding abilities to the key targets of liver cancer (Table 2). The average affinity of EGFR to the top 10 active compounds was −8.8 kcal/mol, which was the lowest among all top 10 targets. Similarly, the average affinity of chrysin (pme0324) to the top 10 targets was −8.1 kcal/mol, which was the lowest among the top 10 active compounds.

**TABLE 2:** Molecular docking results of potential targets with the potential active compounds of *Cyclocarya paliurus.*

Compounds	Affinity (kcal/mol)								
	SRC	IGF1R	MET	AKT1	MAPK14	IKBKB	MAP2K1	PIK3CA	EGFR
Velutin (pma6558)	−6.5	−7.8	−8.4	−6.2	−8.5	−6.2	−8.7	−8.8	−9
Tricin (pmb2850)	−6.2	−8	−8.6	−6.4	−7.3	−6.5	−8.8	−9.1	−9.2
Luteolin (pme0088)	−6.8	−8.1	−8.5	−6.4	−8.4	−6.7	−9.1	−8.8	−9
Chrysin (pme0324)	−6.6	−8	−9.2	−6.7	−8.6	−6.6	−9	−9	−9.3
Chrysoeriol (pme0363)	−6.6	−7.8	−8.5	−6.1	−8.6	−6.5	−9.1	−8.8	−8.9
Apigenin (pme0379)	−6.6	−7.9	−8.7	−6.4	−8.3	−6.5	−8.9	−8.9	−8.8
Kumatakenin (pme1500)	−6.2	−7.7	−8.3	−6.1	−7.5	−6	−8.7	−8.8	−8.9
Baicalein (pme1510)	−6.6	−8.3	−8.6	−6.8	−8.4	−6.8	−8.8	−8.9	−8.8
Tangeretin (pme1550)	−6	−7.2	−7.7	−5.9	−8.1	−5.9	−8	−7.7	−7.8
Pinocembrin (pme2979)	−5.5	−7.3	−7	−6.5	−7.6	−6.4	−7.7	−7.7	−8.3
3-Hydroxyflavone (pme3134)	−6.3	−7.7	−8.2	−6.2	−7.9	−6.1	−8.3	−9.2	−9
Butin (pme3473)	−5.6	−7.6	−7.6	−6.2	−7.6	−6	−8.1	−8	−8.6
7,4′-Dihydroxyflavone (pme3509)	−6.7	−7.8	−8.6	−6.5	−8.4	−6.4	−9.1	−8.8	−9

**FIGURE 6:**
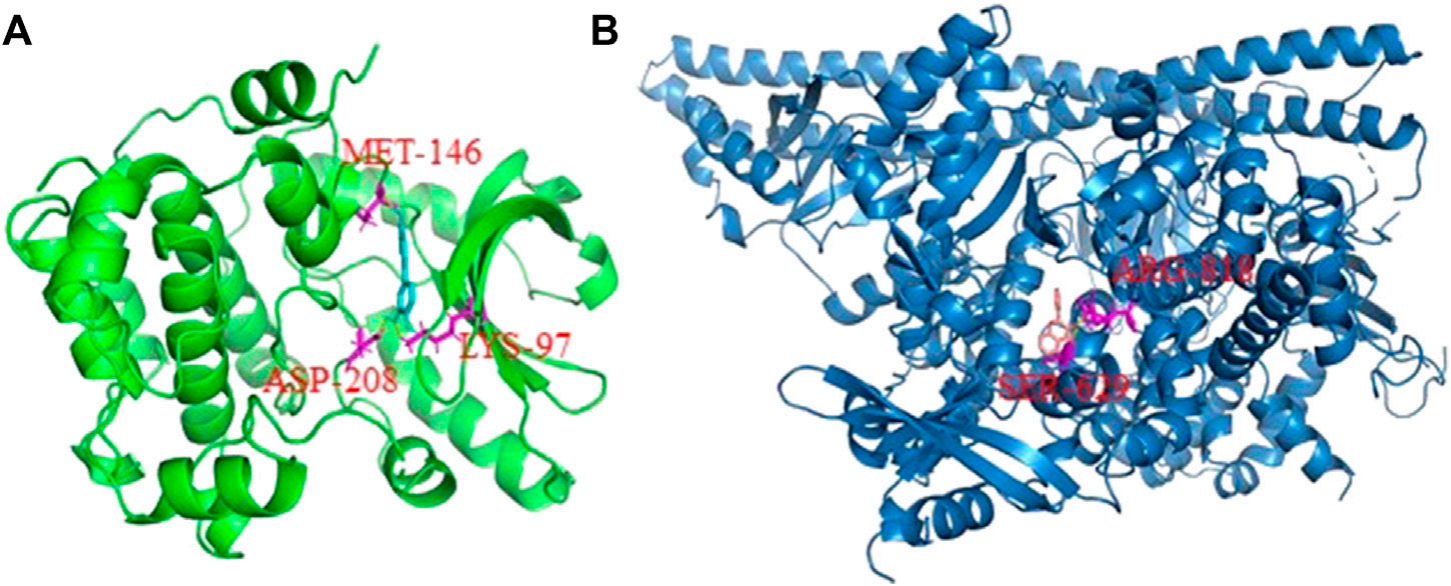
The three-dimensional cartoon display of interactions between active compounds and target receptors. **(A)** MAP2K1 protein with luteolin; **(B)** PIK3CA protein with 3-hydroxyflavone. The amino acid residues which connected with the active compounds with hydron bond were shown in red.

Molecular docking results showed that there were six active compounds with binding energies of lower than −9 kcal/mol to one of the top targets. These included luteolin (pme0088) modeled with MAP2K1 and 3-hydroxyflavone (pme3134) modeled with PIK3CA. Three amino acid residues—namely MET-146, ASP-208 and LYS-97—in MAP2K1 were modeled to form hydrogen bonds with luteolin (Figure 6A). Similarly, ARG-818 and SER-629 of PIK3CA were modeled to form hydrogen bonds with 3-hydroxyflavone (Figure 6B).

### 3.6 *In vitro* verification of two predicted compounds

**FIGURE 7:**
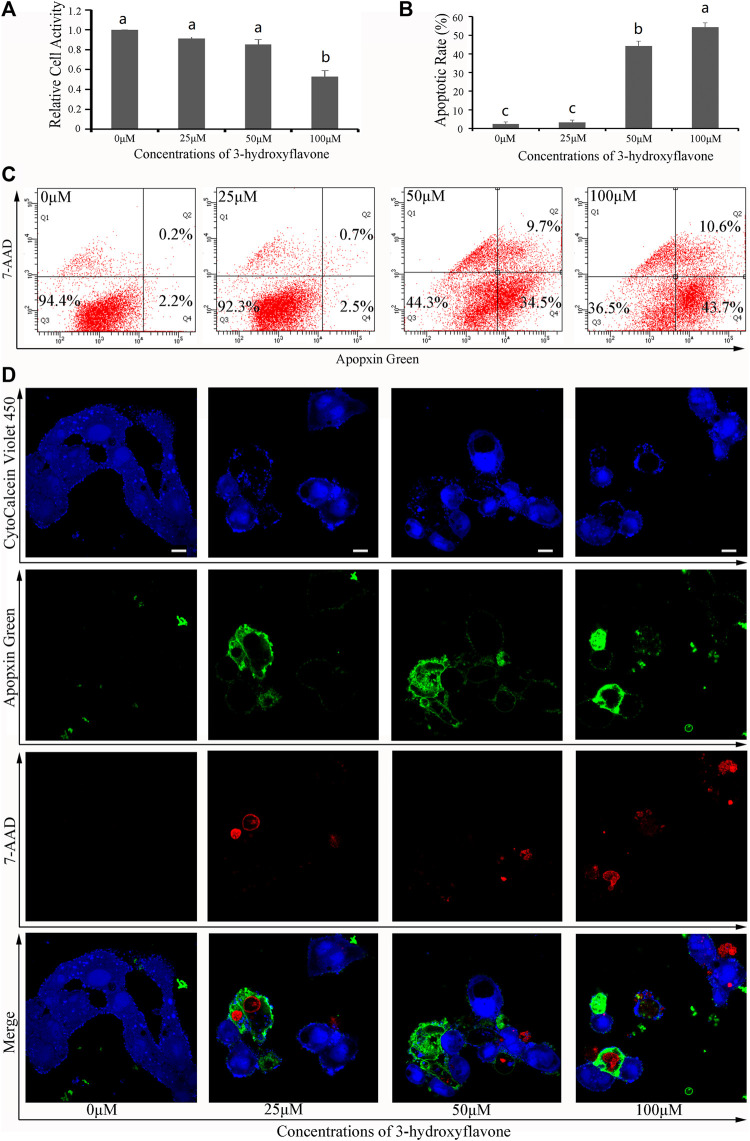
Effects of 3-hydroxyflavone on HepG2 cells. **(A)** Relative cell activity; **(B)** Apoptotic rate. Data are expressed as the mean ± SEM (*n* = 3). Different small letters indicate extremely significant differences between two treatments (*p* < 0.01). **(C)** Representative FACS plots of cells treated with different concentrations of CTFs and stained with Apopxin Green and 7-AAD to identify apoptotic cells in the early and late stages, respectively. From left to right, the concentration of CTFs was 0 μM, 25 μM, 50 μM, and 100 μM. Data are representative of four independent experiments. **(D)** Representative CLSM images of HepG2 cells treated with different concentrations of CTFs and stained with CytoCalcein Violet 450 (in blue color, representing live cells), Apopxin Green (in green color), 7-AAD (in red color). Representative CLSM images of apoptotic HepG2 cells treated with different concentrations of CTFs, CytoCalcein Violet 450 (in blue color, representing live cells), Apopxin Green (in green color), 7-AAD (in red color) and all of three fluorescence stains (merge). From left to right, the concentration of CTFs was 0 μM, 25 μM, 50 μM, and 100 μM. Data are representative of four independent experiments.

**FIGURE 8:**
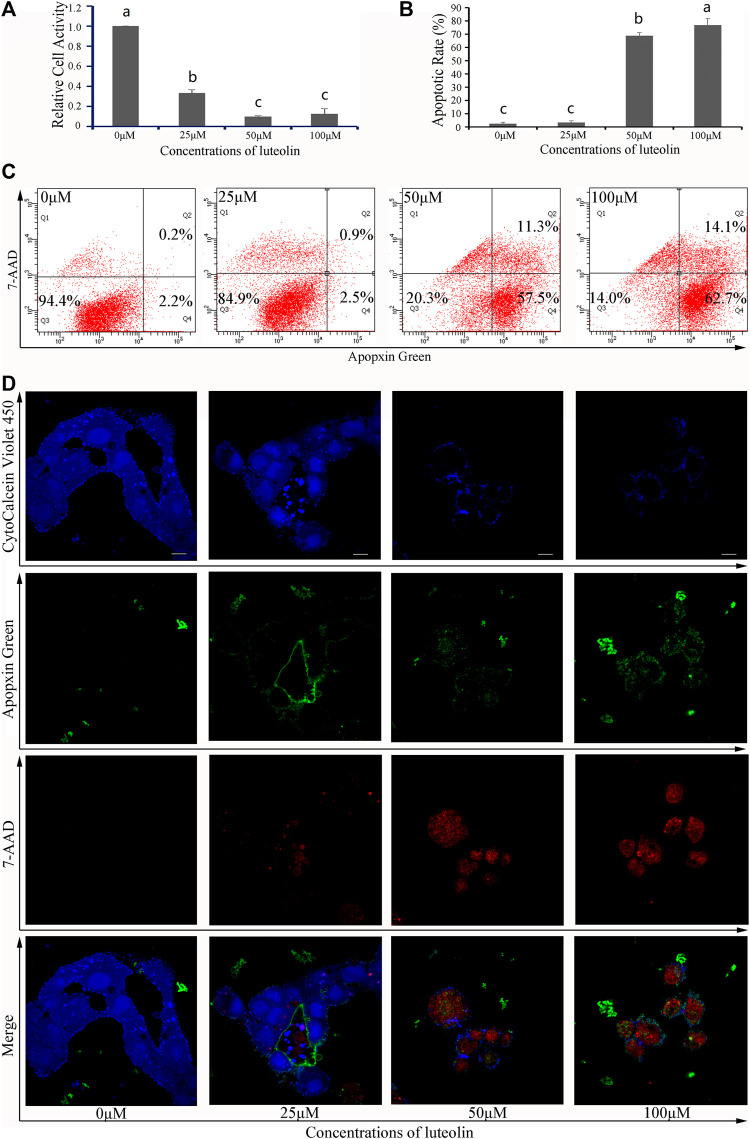
Effects of luteolin on HepG2 cells. **(A)** Relative cell activity; **(B)** Apoptotic rate. Data are expressed as the mean ± SEM (*n* = 3). Different small letters indicate extremely significant differences between two treatments (*p* < 0.01). **(C)** Representative FACS plots of cells treated with different concentrations of CTFs and stained with Apopxin Green and 7-AAD to identify apoptotic cells in the early and late stages, respectively. From left to right, the concentration of CTFs was 0 μM, 25 μM, 50 μM, and 100 μM. Data are representative of four independent experiments. **(D)** Representative CLSM images of HepG2 cells treated with different concentrations of CTFs and stained with CytoCalcein Violet 450 (in blue color, representing live cells), Apopxin Green (in green color), 7-AAD (in red color). Representative CLSM images of apoptotic HepG2 cells treated with different concentrations of CTFs, CytoCalcein Violet 450 (in blue color, representing live cells), Apopxin Green (in green color), 7-AAD (in red color) and all of three fluorescence stains (merge). From left to right, the concentration of CTFs was 0 μM, 25 μM, 50 μM, and 100 μM. Data are representative of four independent experiments.

To validate these results, both 3-hydroxyflavone and luteolin were randomly selected from 13 flaovnoids (Table 1; Figure 5) to inhibit proliferation and induce apoptosis of HepG2 cells *in vitro*. With increasing concentration of both 3-hydroxyflavone and luteolin, the inhibitory effect increased in a dose-dependent manner. When the concentration reached 100 μM, the inhibition rate of 3-hydroxyflavone and luteolin reached 46.71% and 81.49%, respectively (Figures 7A, 8A).

After treatment with 0 μM, 25 μM, 50 μM, and 100 μM of 3-hydroxyflavone for 48 h, the apoptosis rate of HepG2 cells was 5.6% ± 1.1%, 7.7% ± 1.3%, 55.7% ± 2.6%, and 63.5% ± 2.4%, respectively (Figure 7B). After treatment with 0 μM, 25 μM, 50 μM, and 100 μM of luteolin for 48 h, the apoptosis rate of HepG2 cells was 5.6% ± 1.1%, 15.1% ± 1.3%, 79.7% ± 2.3%, and 86% ± 4.9%, respectively (Figure 8B). These indicated that both 3-hydroxyflavone and luteolin could promote the apoptosis of HepG2 cells in a dose-dependent manner (Figures 7C, 8C). Based on the CLSM analysis, cells treated with 25 μM, 50 μM, and 100 μM 3-hydroxyflavone or luteolin led to a corresponding increase of cells in the early and late stages of apoptosis, respectively (Figures 7D, 8D).

## 4 Discussion

In this study, we found that CTFs could significantly inhibit the proliferation of HepG2 cells *in vitro*, and further that the induction of apoptosis might be one of the main mechanisms for the antitumor effect of CTFs. Among the total CTFs, 13 specific flavonoids were identified by network pharmacology as the likely anti-liver cancer compounds, including tangeretin, baicalein, 7,4′-Dihydroxyflavone, velutin, 3-hydroxyflavone, chrysin, kumatakenin, tricin, luteolin, chrysoeriol, apigenin, pinocembrin, and butin. The chemical structures of flavonoids are very similar, with a common diphenylpropane skeleton (C6-C3-C6). The degree of top 13 active flavonoids were ranged from 133 to 144 in present work. Except for 3-Hydroxyflavone (pme3134), the other top 12 active ingredients had hydroxyl or methoxy on their seven position of the diphenylpropane skeleton, indicating the diphenylpropane skeleton was the main active group of flavonoids for anti-tumor effects were also influenced by substituent groups on the diphenylpropane skeleton. Similar studies focused on the anti-liver cancer effects of the flavonoids were performed in traditional Chinese medicines, such as *Peperomia dindygulensis* (Duan et al., 2019). He et al. (2018) also find that *C. paliurus* polysaccharide may show anti-thyroid cancer effect. To the best of our knowledge, this study is the first to report the potential anti-liver cancer effects of CTFs, and indicate likely active ingredients involved in these effects.

Three of these flavonoids—7,4′- dihydroxyflavone, velutin, and butin—were reported for the first time to have anti-cancer activity. Seven flavonoids of these flavonoids- tangeretin, kumatakenin, luteolin, tricin, chrysoeriol, and pinocembrin—have been shown previously to have multiple anti-cancer activity (Table 3), but were shown for the first time here to exhibit specific anti-liver cancer activity. For example, tricin was verified to have anit-tumor effect on prostate cancer PC3 cells (Ghasemi et al., 2018), colorectal tumor (Yue et al., 2020), and other cancer (Chen and Yao, 2013; Li et al., 2021). Four of these flavonoids, i.e., 3-hydroxyflavone, baicalein, chrysin, and apigenin, have been shown to inhibit the proliferation of hepatoma cells as well as those from many other cancers (Table 3). Baicalein, for example, has been shown to exert anti-cancer effects on multiple tumor cell lines, including ovarian cancer cells (Chen J. C. et al., 2013), colorectal cancer cells (Huang et al., 2012), bladder cancer cells (Wu et al., 2013), and breast cancer cells (Liu H. et al., 2016), as well as the liver cancer cell line used in the present study, HepG2 cells (Chen et al., 2009).

**TABLE 3:** Anti-cancer effect of 10 flavonoids.

Flavonoids	Anti-cancer effect	References
Tangeretin	Breast, colon, urinary bladder, lymphoblastoid leukaemia, ovarian, gastric, prostate cancer and leukaemia	Alhamad et al. (2021), Dong et al. (2014), Feng et al. (2016), Guo J. J. et al. (2015), Hirano et al. (1995), Ishii et al. (2010), Lin et al. (2019), Lust et al. (2010), Morley et al. (2007), Raza et al. (2020), Ting et al. (2015), and Zhu et al., 2018
Kumatakenin	Human ovarian cancer cells	Woo et al. (2017)
Luteolin	Prostate, gastric, lung, pancreatic, colon cancer and myeloid leukemia	Imran et al. (2019), Lin et al. (2008), Wu et al. (2008), and Zhou et al. (2009)
Tricin	Prostate, lung, rectal cancer, colorectal tumor	Chen and Yao (2013), Ghasemi et al. (2018), Li et al. (2021), and Yue et al. (2020)
Chrysoeriol	Multiple myeloma, breast cancer	Takemura et al. (2010) and Yang et al. (2010)
Pinocembrin	Prostate, colon cancer and melanoma	Chen Z. et al. (2013), Kumar et al. (2010), and Zheng et al. (2018)
3-Hydroxyflavone	Oral tumor, cervical cancer; adenocarcinoma, and hepatoma	Sakagami et al. (2020) and Lang et al. (2010)
Baicalein	Ovarian, cervical, colorectal, bladde, prostate, breast, ovarian, lung cancer and hepatoma	Chen et al. (2009), Chen J. C. et al. (2013), Guo Z. et al. (2015), Huang et al. (2012), Liu H. et al. (2016), Ma et al. (2016), Peng et al. (2015), Rui et al. (2016), Wu et al. (2013), Yu et al. (2020), and Zheng et al. (2014)
Chrysin	Cervical cancer, leukemia, esophageal squamous carcinoma, malignant glioma, breast carcinoma and prostate cancer, hepatoma	Eaton et al. (1996), Kasala et al. (2015), and Khoo et al. (2010)
Apigenin	Prostate colon, colorectal, gastric, breast, lung, bladder, pancreatic, ovarian, cervical cancer, hepatoma	Eaton et al. (1996), Farah et al. (2003), Kasala et al. (2015), Hu and Cao (2007), Meng et al. (2008), Pan et al. (2013), Ren et al. (2010), Shukla and Gupta (2008), Shukla and Gupta (2010), Wei et al. (2011), Yao (2010); Zhang et al. (2020), and Zhong et al. (2010)

Our analysis identified 10 core target proteins for CTFs in the context of liver cancer, including AKT1, MAPK3, PIK3CA, EGFR, MAP2K1, SRC, IGF1R, IKBKB, MET, and MAPK14. These proteins can be divided into three groups. Proteins in the first group, including AKT1, PIK3CA, MAPK3, MAPK2K1, and MAPK14, have been verified to be involved in the apoptosis of hepatocellular carcinoma cells. In agreement with the existing literature, the present study also identified the PI3K/Akt (hsa04151) and MAPK (hsa04010) signaling pathways as key mechanisms for the potential therapeutic effect of CTFs on liver cancer. The PI3K/AKT/mTOR pathway, which has been previously implicated in hepatocellular carcinoma carcinogenesis (Sun E. J. et al., 2021). Inhibition of PI3K/Akt/mTOR signaling by apigenin and chrysoeriol induces apoptosis and autophagy in hepatocellular carcinoma cells (Yang et al., 2010; Yang et al., 2018). MAPKs also play a key role in intracellular communication, and their activating pathways have been conserved throughout evolution, from plants, fungi, nematodes, and insects, to mammals. MAPK3 and MAPK14 are protein kinases related with cell growth and have been identified previously in many carcinogenesis studies (Baba et al., 2010; Mesquita et al., 2020). There is a strong correlation between MAPK3 and liver cancer resistance (Gao et al., 2016).

The second group of these proteins, including EGFR, IGF1R and MET, represent membrane receptors of various growth factors. EGFR is a rational target for cancer therapy because it is commonly expressed at a high level in a variety of solid tumors and it has been implicated in the control of cell survival, proliferation, metastasis, and angiogenesis (Ciardiello and Tortora, 2003). A significant role for EGFR has been demonstrated in liver regeneration following acute and chronic liver damage, as well as in cirrhosis and hepatocellular carcinoma, highlighting its importance in liver regeneration (Chen et al., 2018). Therefore, blocking EGFR genes might be beneficial to liver cancer treatment. Insulin-like growth factor I receptor (IGF1R) has also been shown to play a critical role in cancer (Peiró et al., 2009), and the inhibition of IGFR1 signaling could prevent prostate cancer cells (Fang et al., 2007). MET, also known as hepatocyte growth factor receptor, is related to biological functions as cell proliferation and progression, apoptosis, metastasis, and morphological changes (Cao et al., 2018). The mutation of MET genes was closely related with multiple cancer, such as hepatocellular carcinomas (Park et al., 1999), and lung cancer (Tsao et al., 1998).

The third group of these proteins, including SRC and IKBKB, represent proteins related with kinases. SRC tyrosine kinases regulate many important mechanisms in both normal and cancerous cells and are overexpressed in a broad range of cancers (Lee and Gautschi, 2006; Zhang and Wu, 2012). IKBKB is one of the most important catalytic subunits of IKK complexes and plays an important regulatory role in activation of NF-KB. The differential expression of IKBKB in human lung adenocarcinoma cells would affect the apoptosis rate (Qi et al., 2014).

In order to verify the accuracy of flavonoids predicted by network pharmacology, we randomly selected two flavonoids to validate *in vitro* with HepG2 cells. Both 3-hydroxyflavone and luteolin were verified to inhibit the proliferation of HepG2 cells in a dose-dependent manner. When the concentration reached 100 μM, the inhibition rate of 3-hydroxyflavone and luteolin reached 46.71% and 81.49%, respectively, while the apoptosis rate of HepG2 cells was 63.5 ± 2.4 and 86 ± 4.9, respectively. For the first time, both of these compounds were shown here to possess anti-liver cancer activity. Little is currently known about 3-hydroxyflavone, while Luteolin (2-[3,4-fihydroxyphenyl]-5,7-dihydroxy-4- chromenone) is one of the most common flavonoids present in edible plants and TCMs (López-Lázaro, 2009). It has been shown to possess multitudinous antioxidant, anti-inflammatory, antimicrobial, and anti-allergic activities (López-Lázaro, 2009). It also possesses anti-cancer activities, such as chemopreventive activity through reduction of DNA damage, mutations, chromosomal aberrations, as well as chemotherapeutic activity through the induction of apoptosis (López-Lázaro, 2009). Fang et al. (2007) found that luteolin inhibits IGFR1 signaling in prostate cancer cells. In this study, we found that the targets of 3-hydroxyflavone and luteolin were MAP2K1 and MET, respectively, while the pathways involved were hsa05163 and hsa01521, respectively. However, the mechanisms underlying the effects of 3-hydroxyflavone and luteolin remain unknown, and additional studies focusing on molecular biology, such as western blotting analysis and molecular interaction analysis, will be needed to more fully elucidate the complex, multifaceted mechanisms by which these compounds exert their anti-cancer effects.

## 5 Conclusion

CTFs have significant inhibitory effects on HepG2 *in vitro* cells by inducing apoptosis. We applied network pharmacology to identify potential key target proteins of CTFs in the context of liver cancer by constructing a target interaction network, and used molecular docking methods to validate the key findings. Our results indicate that 13 flavonoid compounds from the leaves of *C. paliurus* have potential anti-liver cancer activity, including tangeretin, baicalein, 7,4′-dihydroxyflavone, velutin, 3-hydroxyflavone, chrysin, kumatakenin, tricin, luteolin, chrysoeriol, apigenin, pinocembrin, and butin. These flavonoids were predicted to interact with AKT1, MAPK3, PIK3CA, EGFR, MAP2K1, SRC, IGF1R, IKBKB, MET, and MAPK14. KEGG pathway enrichment analysis revealed that *C. paliurus* may exert its inhibitory effect on hepatocellular carcinoma by regulating pathways related to cancer (hsa05200), PI3K-Akt signaling pathway (hsa04151), proteoglycans in cancer pathway (hsa05205), microRNAs in cancer pathway (hsa05206), and endocrine resistance pathway (hsa01522) *via* core target proteins. Both 3-hydroxyflavone and luteolin were verified to inhibit the proliferation of HepG2 cells *in vitro* by inducing apoptosis. Although our results were obtained by network pharmacology and more experiments should be conducted to make it more confidence, our study provides scientific evidence supporting the use of CTFs for the treatment of liver cancer. Furthermore, we should realized that the low water-solubility of the flavonoids might limit the application of flavonoids in *in-vivo* cancer treatment, however, it may be overcome by different pharmaceutical methods, such as self-nanoemulsion drug delivery system and phospholipid complex.

## Data Availability

The original contributions presented in the study are included in the article/Supplementary Material, further inquiries can be directed to the corresponding authors.
