# Supplementary material for: The mechanism of flavonoids from Cyclocarya paliurus on inhibiting liver cancer based on in vitro experiments and network pharmacology
Source: Front Pharmacol. 2023 Feb 3;14:1049953. doi: 10.3389/fphar.2023.1049953 (PMC9936097; doi:10.3389/fphar.2023.1049953)
Supplement: Supplementary file 1 [file DataSheet1.docx]

Table S1 The information of 62 flavonoids compounds in *Cyclocarya paliurus*

| Mol ID | Compounds | Degree | Betweenness centrality | Molecular Formula | CAS |
| --- | --- | --- | --- | --- | --- |
| pmb0580 | Chrysin 5-O-glucoside (Toringin) | 89 | 0.005415036 | C21H20O9 | 1329-10-8 |
| pme0324 | Chrysin | 136 | 0.013738984 | C15H10O4 | 480-40-0 |
| pme0379 | Apigenin | 133 | 0.009923388 | C15H10O5 | 520-36-5 |
| pme1601 | Apigenin 4-O-rhamnoside | 88 | 0.005270951 | C21H20O9 | 133538-77-9 |
| pme0363 | Chrysoeriol | 133 | 0.009729275 | C16H12O6 | 491-71-4 |
| pme2319 | Hesperetin | 119 | 0.017068352 | C16H14O6 | 69097-99-0 |
| pma1116 | Kaempferide | 130 | 0.009041031 | C16H12O6 | 491-54-3 |
| pme0196 | Kaempferol | 132 | 0.009435457 | C15H10O6 | 520-18-3 |
| pme2963 | Aromadedrin (Dihydrokaempferol) | #N/A | #N/A | C15H12O6 | 5150-32-3 |
| pme0088 | Luteolin | 133 | 0.009923388 | C15H10O6 | 491-70-3 |
| pme0376 | Naringenin | 132 | 0.018739359 | C15H12O5 | 67604-48-2 |
| pme2957 | Naringenin chalcone | 128 | 0.010697844 | C15H12O5 | 73692-50-9 |
| pme0199 | Quercetin | 130 | 0.009026014 | C15H10O7 | 117-39-5 |
| pme1521 | Dihydroquercetin (Taxifolin) | #N/A | #N/A | C15H12O7 | 98006-93-0 |
| pme3288 | 3,7-Di-O-methylquercetin | 131 | 0.009475901 | C17H14O7 | 2068-02-2 |
| pme3369 | Rhamnetin (7-O-methxyl quercetin) | 131 | 0.009475901 | C16H12O7 | 90-19-7 |
| pme3300 | Tricetin | 131 | 0.009196812 | C15H10O7 | 520-31-0 |
| pme1568 | Orobol (5,7,3',4'-tetrahydroxyisoflavone) | 118 | 0.011791011 | C15H10O6 | 480-23-9 |
| pme3134 | 3-Hydroxyflavone | 137 | 0.013649042 | C15H10O3 | 577-85-5 |
| pmc1990 | 4'-Hydroxy-5,7-dimethoxyflavanone | 122 | 0.017420722 | C17H16O5 | 26207-67-0 |
| pme3509 | 7,4'-Dihydroxyflavone | 140 | 0.015987649 | C15H10O4 | 2196-14-7 |
| pme0205 | Catechin | #N/A | #N/A | C15H14O6 | 7295-85-4 |
| pme0450 | L-Epicatechin | #N/A | #N/A | C15H14O6 | 7295-85-4 |
| pme1544 | Acacetin | 118 | 0.009142903 | C16H12O5 | 480-44-4 |
| pme1510 | Baicalein (5,6,7-Trihydroxyflavone) | 141 | 0.016154586 | C15H10O5 | 491-67-8 |
| pme1588 | Isorhamnetin | 131 | 0.009312151 | C16H12O7 | 480-19-3 |
| pmb0745 | Tricin 4'-O-syringyl alcohol | 112 | 0.006916282 | C26H24O10 |  |
| pmb2850 | Tricin | 133 | 0.010072666 | C17H14O7 | 520-32-1 |
| pme3276 | 2'-Hydroxygenistein | 103 | 0.006432961 | C15H10O6 | 1156-78-1 |
| pme1580 | Eriodictyol | 129 | 0.013332311 | C15H12O6 | 4049-38-1 |
| pme1599 | 7-O-Methyleriodictyol | 87 | 0.005956367 | C16H14O6 | 51857-11-5 |
| pme3461 | Homoeriodictyol | 92 | 0.006810798 | C16H14O6 | 107657-60-3 |
| pme1662 | sakuranetin | 85 | 0.007458304 | C16H14O5 | 520-29-6 |
| pme3464 | Isosakuranetin (4'-Methylnaringenin) | 127 | 0.013674433 | C16H14O5 | 480-43-3 |
| pmb0563 | Peonidin | 129 | 0.009748776 | C16H13O6 | 134-01-0 |
| pme3401 | Syringetin | 129 | 0.008870532 | C17H14O8 | 4423-37-4 |
| pme1824 | Protocatechuic acid | 43 | 0.001034938 | C7H6O4 | 99-50-3 |
| pme2478 | Protocatechuic aldehyde | #N/A | #N/A | C7H6O3 | 139-85-5 |
| pma6389 | Ayanin | 132 | 0.009672255 | C18H16O7 | 572-32-7 |
| pma6558 | Velutin | 139 | 0.014812507 | C17H14O6 | 25739-41-7 |
| pma6576 | Spinacetin | 131 | 0.009475901 | C17H14O8 | 3153-83-1 |
| pme0355 | Daidzein | 80 | 0.00516935 | C15H10O4 | 486-66-8 |
| pme1201 | Phloretin | 98 | 0.010333804 | C15H14O5 | 60-82-2 |
| pme1397 | Pelargonidin | 128 | 0.009581842 | C15H11O5 | 7690-51-9 |
| pme1399 | Xanthohumol | 80 | 0.003952148 | C21H22O5 | 569-83-5 |
| pme1500 | Kumatakenin | 135 | 0.011668801 | C17H14O6 | 3301-49-3 |
| pme1518 | Nobiletin | 35 | 0.000617852 | C21H22O8 | 478-01-3 |
| pme1550 | Tangeretin | 144 | 0.019500038 | C20H20O7 | 481-53-8 |
| pme2979 | Pinocembrin (Dihydrochrysin) | 133 | 0.026505687 | C15H12O4 | 68745-38-0 |
| pme3230 | Calycosin | 74 | 0.003721688 | C16H12O5 | 20575-57-9 |
| pme3250 | Biochanin A | 69 | 0.002848058 | C16H12O5 | 491-80-5 |
| pme3261 | 6-Hydroxydaidzein | 95 | 0.006324627 | C15H10O5 | 17817-31-1 |
| pme3282 | Afzelechin (3,5,7,4'-Tetrahydroxyflavan) | #N/A | #N/A | C15H14O5 | 490-61-9 |
| pme3292 | Prunetin | 69 | 0.002848058 | C16H12O5 | 552-59-0 |
| pme3396 | Fustin | #N/A | #N/A | C15H12O6 | 20725-03-5 |
| pme3400 | Sissotrin | 51 | 0.001939282 | C22H22O10 | 5928-26-7 |
| pme3410 | Laricitrin | 131 | 0.009312151 | C16H12O8 | 53472-37-0 |
| pme3439 | Butein | 112 | 0.011604528 | C15H12O5 | 21849-70-7 |
| pme3451 | Rotenone | #N/A | #N/A | C23H22O6 | 12679-58-2 |
| pme3473 | Butin | 133 | 0.017930546 | C15H12O5 | 21913-99-5 |
| pme3502 | Formononetin 7-O-glucoside (Ononin) | 93 | 0.007762707 | C22H22O9 | 486-62-4 |
| pme3514 | Morin | 114 | 0.00702026 | C15H10O7 | 480-16-0 |

Table S2 The information of the target proteins used in the manuscript

| UniProt ID | Protein | Gene | Betweenness centrality | Degree |
| --- | --- | --- | --- | --- |
| P98170 | Inhibitor of apoptosis protein 3 | XIAP | 7.67E-05 | 12 |
| P15692 | Vascular endothelial growth factor A | VEGFA | 2.05E-04 | 27 |
| P01375 | TNF-alpha | TNF | 0.001036182 | 46 |
| O14746 | Telomerase reverse transcriptase | TERT | 3.37E-04 | 40 |
| P42224 | Signal transducer and activator of transcription 1-alpha/beta | STAT1 | 4.50E-04 | 31 |
| P12931 | Tyrosine-protein kinase SRC | SRC | 7.41E-04 | 68 |
| Q96EB6 | NAD-dependent deacetylase sirtuin 1 | SIRT1 | 2.62E-05 | 11 |
| P19793 | Retinoid X receptor alpha | RXRA | 1.65E-04 | 24 |
| Q05397 | Focal adhesion kinase 1 | PTK2 | 3.30E-04 | 48 |
| P35354 | Cyclooxygenase-2 | PTGS2 | 2.74E-04 | 36 |
| P37231 | Peroxisome proliferator-activated receptor gamma | PPARG | 9.25E-05 | 19 |
| Q07869 | Peroxisome proliferator-activated receptor alpha | PPARA | 3.17E-05 | 13 |
| P00749 | Urokinase-type plasminogen activator | PLAU | 2.27E-05 | 11 |
| Q9P1W9 | Serine/threonine-protein kinase PIM2 | PIM2 | 1.27E-06 | 3 |
| P48736 | PI3-kinase p110-gamma subunit | PIK3CG | 1.38E-04 | 32 |
| P42336 | PI3-kinase p110-alpha subunit | PIK3CA | 0.004255044 | 93 |
| P09874 | Poly [ADP-ribose] polymerase-1 | PARP1 | 1.10E-04 | 25 |
| P35228 | Nitric oxide synthase, inducible | NOS2 | 1.56E-04 | 26 |
| P42345 | Serine/threonine-protein kinase mTOR | MTOR | 0.001090103 | 47 |
| P14780 | Matrix metalloproteinase 9 | MMP9 | 4.15E-04 | 52 |
| P09237 | Matrix metalloproteinase 7 | MMP7 | 0 | 2 |
| P08253 | Matrix metalloproteinase 2 | MMP2 | 3.82E-04 | 48 |
| P08581 | Hepatocyte growth factor receptor | MET | 4.39E-04 | 56 |
| Q07820 | Induced myeloid leukemia cell differentiation protein Mcl-1 | MCL1 | 1.09E-04 | 24 |
| P27361 | MAP kinase ERK1 | MAPK3 | 0.005326769 | 96 |
| Q16539 | MAP kinase p38 alpha | MAPK14 | 0.00146239 | 53 |
| Q02750 | Dual specificity mitogen-activated protein kinase kinase 1 | MAP2K1 | 0.003000439 | 77 |
| P10721 | Stem cell growth factor receptor | KIT | 2.28E-04 | 32 |
| P35968 | Vascular endothelial growth factor receptor 2 | KDR | 3.47E-04 | 47 |
| P23458 | Tyrosine-protein kinase JAK1 | JAK1 | 4.08E-04 | 27 |
| P60568 | Interleukin-2 | IL2 | 1.43E-04 | 23 |
| O14920 | Inhibitor of nuclear factor kappa B kinase beta subunit | IKBKB | 0.001871632 | 60 |
| P08069 | Insulin-like growth factor I receptor | IGF1R | 6.42E-04 | 62 |
| P41235 | Hepatocyte nuclear factor 4-alpha | HNF4A | 3.97E-06 | 5 |
| Q16665 | Hypoxia-inducible factor 1 alpha | HIF1A | 7.16E-05 | 14 |
| P11362 | Fibroblast growth factor receptor 1 | FGFR1 | 8.57E-05 | 20 |
| P00734 | Thrombin | F2 | 1.45E-04 | 32 |
| P03372 | Estrogen receptor alpha | ESR1 | 4.93E-04 | 44 |
| P04626 | Receptor protein-tyrosine kinase erbB-2 | ERBB2 | 1.62E-04 | 22 |
| P00533 | Epidermal growth factor receptor erbB1 | EGFR | 0.001280438 | 85 |
| P26358 | DNA (cytosine-5)-methyltransferase 1 | DNMT1 | 4.28E-06 | 5 |
| P05177 | Cytochrome P450 1A2 | CYP1A2 | 6.98E-06 | 8 |
| P04798 | Cytochrome P450 1A1 | CYP1A1 | 1.08E-05 | 10 |
| P61073 | C-X-C chemokine receptor type 4 | CXCR4 | 1.97E-05 | 8 |
| P25024 | Interleukin-8 receptor A | CXCR1 | 1.17E-04 | 28 |
| P11802 | Cyclin-dependent kinase 4 | CDK4 | 2.75E-04 | 29 |
| P24941 | Cyclin-dependent kinase 2 | CDK2 | 3.70E-04 | 49 |
| P06493 | Cyclin-dependent kinase 1 | CDK1 | 2.21E-04 | 38 |
| P30304 | Dual specificity phosphatase Cdc25A | CDC25A | 5.50E-06 | 5 |
| Q07817 | Apoptosis regulator Bcl-X | BCL2L1 | 2.61E-04 | 26 |
| P10415 | Apoptosis regulator Bcl-2 | BCL2 | 6.75E-04 | 42 |
| O14965 | Serine/threonine-protein kinase Aurora-A | AURKA | 7.41E-06 | 7 |
| P05089 | Arginase-1 (by homology) | ARG1 | 1.76E-04 | 30 |
| P10275 | Androgen Receptor | AR | 2.98E-05 | 15 |
| P05091 | Aldehyde dehydrogenase | ALDH2 | 1.16E-04 | 16 |
| P31749 | Serine/threonine-protein kinase AKT | AKT1 | 0.004041549 | 113 |
| Q9UNQ0 | ATP-binding cassette sub-family G member 2 | ABCG2 | 9.08E-04 | 49 |
| P33527 | Multidrug resistance-associated protein 1 | ABCC1 | 5.26E-04 | 46 |
| P08183 | P-glycoprotein 1 | ABCB1 | 6.76E-04 | 47 |

Table S3. The docking results of top 10 targets with top 10 active compounds

| Compounds | Affinity (kcal/mol) | | | | | | | | |
| --- | --- | --- | --- | --- | --- | --- | --- | --- | --- |
|  | SRC | IGF1R | MET | AKT1 | MAPK14 | IKBKB | MAP2K1 | PIK3CA | EGFR |
| pma6558 | -6.5 | -7.8 | -8.4 | -6.2 | -8.5 | -6.2 | -8.7 | -8.8 | -9 |
| pmb2850 | -6.2 | -8 | -8.6 | -6.4 | -7.3 | -6.5 | -8.8 | -9.1 | -9.2 |
| pme0088 | -6.8 | -8.1 | -8.5 | -6.4 | -8.4 | -6.7 | -9.1 | -8.8 | -9 |
| pme0324 | -6.6 | -8 | -9.2 | -6.7 | -8.6 | -6.6 | -9 | -9 | -9.3 |
| pme0363 | -6.6 | -7.8 | -8.5 | -6.1 | -8.6 | -6.5 | -9.1 | -8.8 | -8.9 |
| pme0379 | -6.6 | -7.9 | -8.7 | -6.4 | -8.3 | -6.5 | -8.9 | -8.9 | -8.8 |
| pme1500 | -6.2 | -7.7 | -8.3 | -6.1 | -7.5 | -6 | -8.7 | -8.8 | -8.9 |
| pme1510 | -6.6 | -8.3 | -8.6 | -6.8 | -8.4 | -6.8 | -8.8 | -8.9 | -8.8 |
| pme1550 | -6 | -7.2 | -7.7 | -5.9 | -8.1 | -5.9 | -8 | -7.7 | -7.8 |
| pme2979 | -5.5 | -7.3 | -7 | -6.5 | -7.6 | -6.4 | -7.7 | -7.7 | -8.3 |
| pme3134 | -6.3 | -7.7 | -8.2 | -6.2 | -7.9 | -6.1 | -8.3 | -9.2 | -9 |
| pme3473 | -5.6 | -7.6 | -7.6 | -6.2 | -7.6 | -6 | -8.1 | -8 | -8.6 |
| pme3509 | -6.7 | -7.8 | -8.6 | -6.5 | -8.4 | -6.4 | -9.1 | -8.8 | -9 |


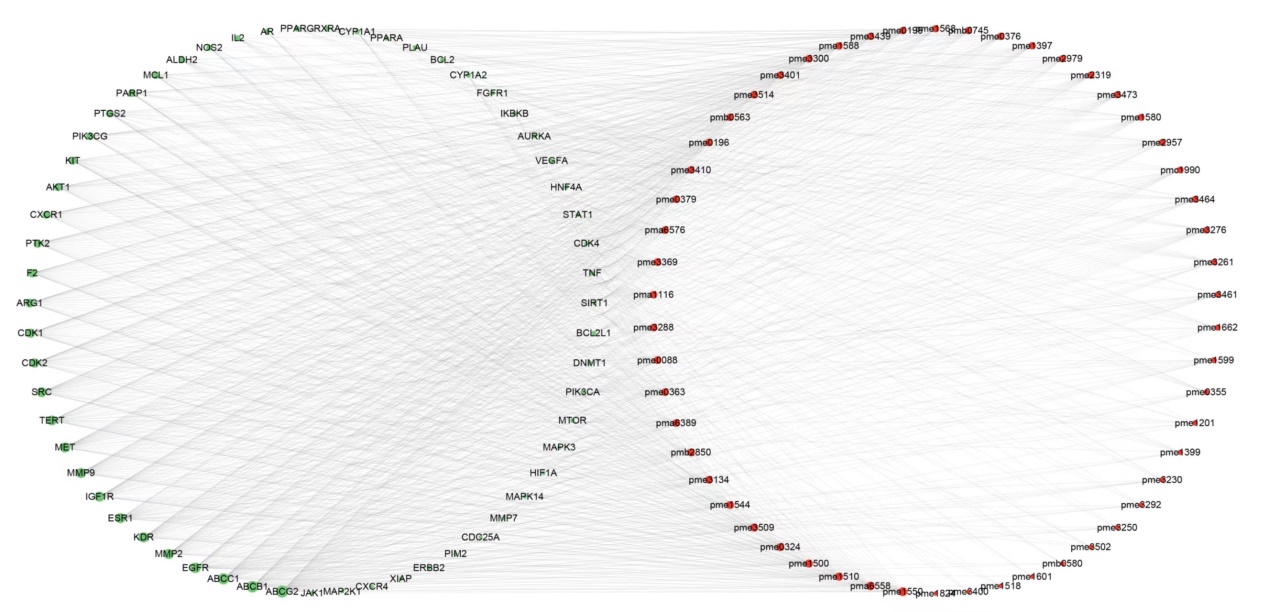


Fig. S1 *Cyclocarya paliurus*'s component-target network. The red node represents the active ingredients, the green node represents the target proteins, and the size of the nodes represents the size of the degree value.


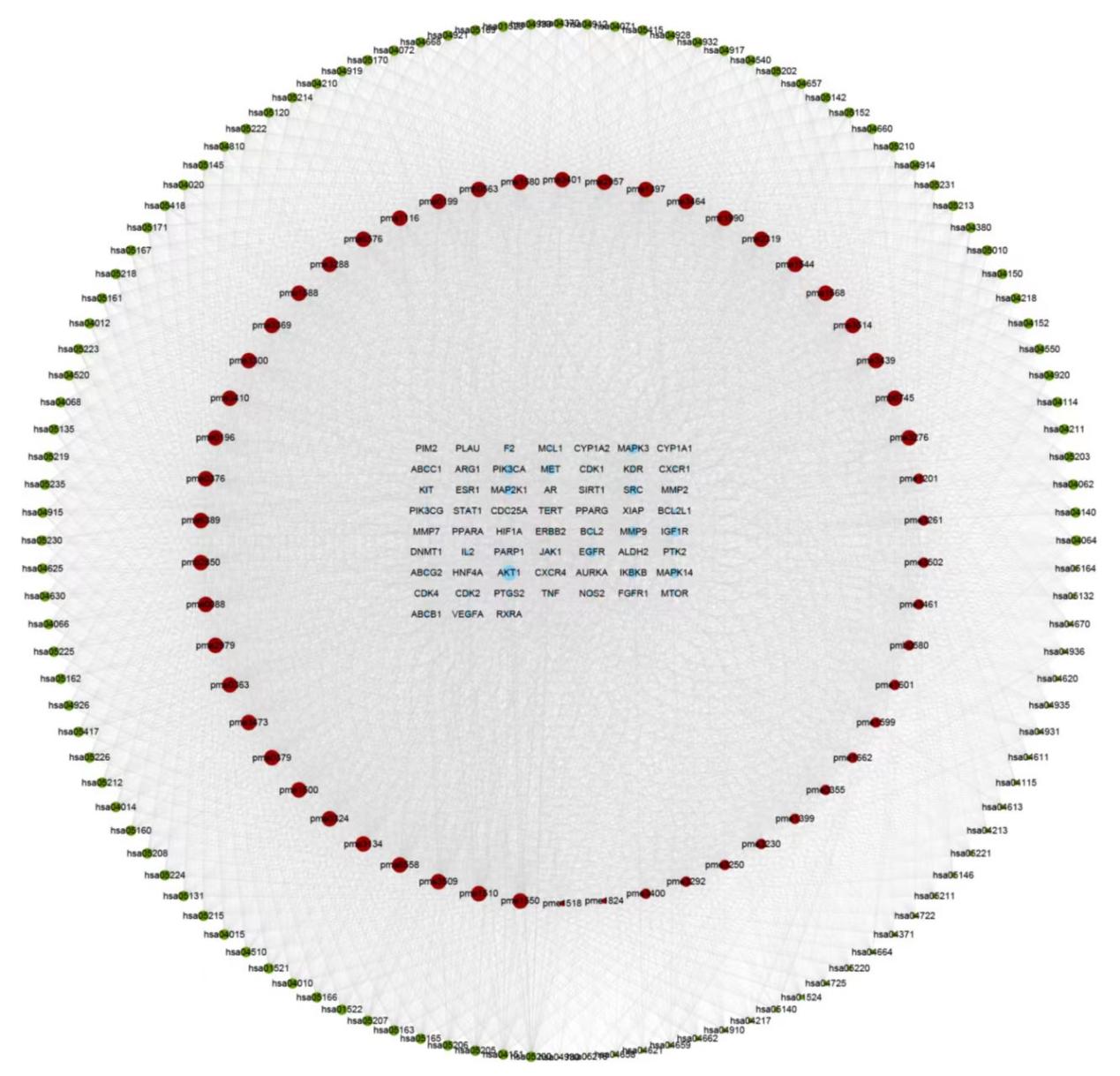


Fig. S2 *Cyclocarya paliurus*'s component-target-pathway network. The red node represents the active ingredient, the blue node represents the target, the green node represents the pathway, and the size of the node represents of the degree value. Lines represent the relationships between the compounds, targets, and pathways.
